# How Ionization Catalyzes Diels‐Alder Reactions

**DOI:** 10.1002/chem.202200987

**Published:** 2022-05-13

**Authors:** Pascal Vermeeren, Trevor A. Hamlin, F. Matthias Bickelhaupt

**Affiliations:** ^1^ Department of Theoretical Chemistry Amsterdam Institute of Molecular and Life Sciences (AIMMS) Amsterdam Center for Multiscale Modeling (ACMM) Vrije Universiteit Amsterdam De Boelelaan 1083 1081 HV Amsterdam The Netherlands; ^2^ Institute for Molecules and Materials Radboud University Nijmegen Heyendaalseweg 135 6525 AJ Nijmegen The Netherlands

**Keywords:** activation strain model, density functional calculations, Diels-Alder reaction, radical chemistry, reactivity

## Abstract

The catalytic effect of ionization on the Diels‐Alder reaction between 1,3‐butadiene and acrylaldehyde has been studied using relativistic density functional theory (DFT). Removal of an electron from the dienophile, acrylaldehyde, significantly accelerates the Diels‐Alder reaction and shifts the reaction mechanism from concerted asynchronous for the neutral Diels‐Alder reaction to stepwise for the radical‐cation Diels‐Alder reaction. Our detailed activation strain and Kohn‐Sham molecular orbital analyses reveal how ionization of the dienophile enhances the Diels‐Alder reactivity via two mechanisms: (i) by amplifying the asymmetry in the dienophile's occupied π‐orbitals to such an extent that the reaction goes from concerted asynchronous to stepwise and thus with substantially less steric (Pauli) repulsion per reaction step; (ii) by enhancing the stabilizing orbital interactions that result from the ability of the singly occupied molecular orbital of the radical‐cation dienophile to engage in an additional three‐electron bonding interaction with the highest occupied molecular orbital of the diene.

## Graphical abstract

**Let's get radical**! Quantum chemical activation strain analyses reveal how ionization of a dienophile can effectively catalyze the Diels‐Alder reaction by reducing the Pauli repulsion and simultaneously enhancing the orbital interactions. This study nicely demonstrates the similarities and differences between radical‐cation and Lewis acid‐catalyzed Diels‐Alder reactions.

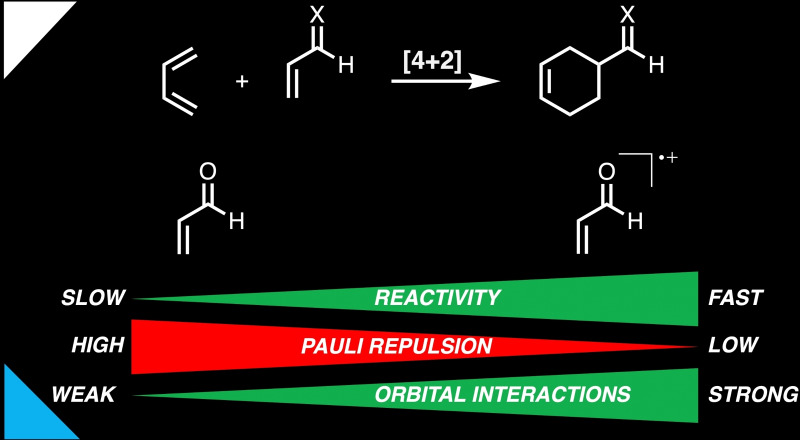

## Introduction

More than ninety years after its discovery, the Diels‐Alder (DA) cycloaddition reaction is still one of the most important chemical transformations in synthetic organic chemistry.^[1]^ It is an effective strategy for synthesizing densely functionalized six‐membered rings, with up to four stereocenters, in a single reaction step with high atom efficiency.[^2^, ^3^] As a result, this organic reaction has been widely utilized to synthesize many target compounds, including complex natural products, bioactive molecules, and polymer materials.[^4^, ^5^]

A convenient method to catalyze the DA reaction is by coordinating a Lewis acid to the dienophile.^[6]^ Until recently, it was commonly accepted that this enhanced DA reactivity originates from lowering the LUMO of the activated dienophile, which, ultimately, gives rise to a smaller normal electron demand (NED) HOMO_diene_–LUMO_dienophile_ orbital energy gap and hence more stabilizing orbital interactions.^[7]^ Recently, however, we discovered that this rationale behind the enhanced reactivity of Lewis acid‐catalyzed DA reactions is incorrect.^[8]^ Lewis acids not only lower the energy of the LUMO_dienophile_ and strengthen the NED orbital interaction, but also lower the energy of the HOMO_dienophile_ and hence weaken the inverse electron demand (IED) LUMO_diene_–HOMO_dienophile_ orbital interaction. The strengthening of the NED gets effectively counteracted by the weakening of the IED orbital interactions, resulting in a similar orbital interaction for both the uncatalyzed and LA‐catalyzed DA reaction.

Instead, the enhanced DA reactivity originates from a significant reduction of destabilizing two‐center four‐electron Pauli repulsion between the occupied π‐molecular orbitals of the reactants. The LA binds via a donor‐acceptor interaction to the dienophile which reduces the amplitude of its occupied π‐orbital by moving part of the amplitude to the LA. The reduced π‐electron amplitude on the dienophile‘s reactive C=C bond, in turn, results in a reduced closed‐shell–closed‐shell orbital overlap with the incoming diene and thus to a less destabilizing Pauli repulsion. In addition, the LA also increases the asynchronicity of the DA reaction by introducing an asymmetry in the π‐orbital on the reactive C=C double bond of the dienophile.^[9]^ This enhanced asynchronicity leads to both an additional lowering of the Pauli repulsion and less pressure on the reactants to deform, *i. e*., less destabilizing activation strain, and thus an additional lowering of the reaction barrier. Besides LA‐catalyzed Diels‐Alder reaction, we, as well as others,^[10]^ have found that this mode of catalysis is also active in a variety of other catalyzed organic reactions,^[11]^ such as, among others, aza‐Michael addition reactions^[11a]^ and ene reactions.^[11d]^ We have coined the concept of *Pauli‐lowering catalysis* to refer to this apparently ubiquitous electronic mechanism behind LA catalysis.^[12]^

The most extreme case of pulling electrons from the dienophile is ionization, which turns a closed‐shell dienophile into a radical‐cation dienophile. The latter, in turn, can engage in a radical‐cation Diels‐Alder reaction.^[13]^ The concept of radical‐cation Diels‐Alder reactions has been extensively employed in organic synthesis to accelerate thermally not accessible neutral Diels‐Alder reactions.^[14]^ Previous theoretical studies revealed that radical‐cation Diels‐Alder reactions proceed via a stepwise reaction mechanism, not a concerted one as commonly observed for neutral, closed‐shell Diels‐Alder reactions.^[15]^ In the stepwise mechanism, the diene and dienophile first form one new carbon−carbon bond, resulting in an acyclic intermediate, in which the two original reactants are in an *anti*‐conformation, followed by a cyclization step, during which the original reactants rotate around the newly formed C−C bond from an *anti*‐ to a *gauche*‐conformation and the second carbon−carbon bond is made, yielding the six‐membered cycloadduct. Depending on the reactants, the cyclization step goes via one transition state, where both rotation and bond formation happen simultaneously, or via two transition states, where the rotation and bond formation occur sequentially. The increased reaction rate for radical‐cation Diels‐Alder reactions is currently ascribed to the fact that the transition state of the first, and rate‐determining, reaction step is much more reactant‐like than the analogous neutral Diels‐Alder reaction between two closed‐shell reactants and the reaction barrier will, therefore, be significantly lower.^[13]^ Despite advances on understanding radical‐cation Diels‐Alder reactions, little quantitative data is available regarding the physical origin of the rate enhancement of radical‐cation Diels‐Alder reactions.

**Scheme 1:**
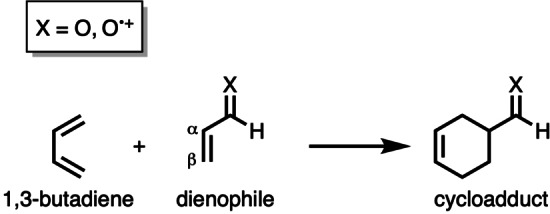
The uncatalyzed and radical‐cation Diels‐Alder reactions between 1,3‐butadiene (**B**) and α,β‐unsaturated dienophile (**X**) that were computationally studied.

In this work, we have performed a detailed computational study to disentangle the physical mechanism behind the rate enhancement of Diels‐Alder reactions upon ionization of the dienophile. To this end, we have analyzed and compared the neutral Diels‐Alder reaction between 1,3‐butadiene (**B**), acting as a diene, and an α,β‐unsaturated dienophile (**X**) with the radical‐cation Diels‐Alder reaction involving an ionized dienophile, using relativistic density functional theory (DFT) at ZORA‐(U)BP86/TZ2P (Scheme 1). These reactants were selected in order to directly compare the results obtained in this work with our previous studies on Lewis acid‐catalyzed Diels‐Alder reactions,[^8a^, ^8b^, ^8c^, ^8d^] which enables us to find similarities and differences between radical‐cation Diels‐Alder reactions and Lewis acid‐catalyzed Diels‐Alder reactions. The activation strain model (ASM)^[16]^ of reactivity in combination with Kohn‐Sham molecular orbital (KS‐MO)[^17^, ^18^] theory and a matching canonical energy decomposition analysis (EDA)^[19]^ were employed to obtain quantitative insights into the origin of the enhanced reactivity of radical‐cation Diels‐Alder reactions. This methodology has proven to be reliable for the understanding of fundamental processes in organic chemistry, such as cycloaddition reactions.[^8^, ^11^]

## Computational Method

### Computational details

All calculations were performed using the Amsterdam Density Functional (ADF2019.102) software package.^[20]^ The generalized gradient approximation (GGA) functional BP86 at the spin‐unrestricted formalism was used for the optimizations of all stationary points as well as the analyses.^[21]^ The basis set employed, denoted TZ2P,^[22]^ is of triple‐ζ quality and is augmented with two sets of polarization functions on each atom. Scalar relativistic effects are accounted for using the zeroth‐order regular approximation (ZORA).^[23]^ This level of theory, *i. e*., ZORA‐(U)BP86/TZ2P, has been proven to be accurate in calculating the relative trends in reaction barriers and energies as well as performing the activation strain and energy decomposition analyses for cycloaddition reactions,[^8^, ^11^, ^24^] and gives a nearly negligible spin contamination for the radical‐cation species, with ⟨*S* ^2^⟩ values that are close to the ⟨*S* ^2^⟩=0.75 that is expected for doublet radicals (See Table S1).^[25]^ The accuracies of the fit scheme (Zlm fit)^[26a]^ and the integration grid (Becke grid)^[26b]^ were set to VERYGOOD. Equilibrium and transition state geometries were verified by means of vibrational analyses,^[27]^ to assess the nature of all structures: for minima, no imaginary frequencies were found, whereas transition states present a single imaginary frequency. Besides, the character of the normal mode associated with the imaginary frequency was analyzed to ensure that the correct transition state was found. The potential energy surfaces of the studied Diels‐Alder reactions were obtained by utilizing intrinsic reaction coordinate (IRC) calculations.^[28]^ The acquired potential energy surfaces were analyzed using the PyFrag 2019 program.^[29]^ Optimized structures were illustrated using CYLview.^[30]^

### Activation strain model and energy decomposition analysis

The activation strain model of chemical reactivity (ASM,^[16]^ also known as the distortion/interaction model^[31]^), is a fragment‐based approach based on the idea that the energy of a reacting system, *i. e*., the potential energy surface, is described with respect to, and understood in terms of the characteristics of, the original reactants, which are the 1,3‐butadiene (**B**) and α,β‐unsaturated dienophile (**X**). It considers their rigidity and the extent to which the reactants must deform during the reaction plus their capability to interact as the reaction proceeds. In this model, we decompose the total energy, Δ*E*(ζ), into the strain and interaction energy, Δ*E*_strain_(ζ) and Δ*E*_int_(ζ), respectively, along the IRC which is projected onto a reaction coordinate ζ that is critically involved in the reaction [Eq. 1].

(1) $$\Delta E\left(\zeta \right)=\Delta E{}_{\mathrm{strain}}\left(\zeta \right)+\Delta E{}_{\mathrm{int}}\left(\zeta \right)$$

In this equation, the strain energy, Δ*E*_strain_(ζ), is the penalty that needs to be paid to deform the reactants from their equilibrium structure to the geometry they adopt during the reaction at point ζ of the reaction coordinate. On the other hand, the interaction energy, Δ*E*_int_(ζ), accounts for all the mutual interactions that occur between the deformed fragments along the reaction coordinate.

The interaction energy between the deformed reactants is further analyzed by means of our canonical energy decomposition analysis (EDA) scheme.^[19]^ The EDA decomposes the Δ*E*_int_(ζ) into the following three physically meaningful energy terms [Eq. 2]:

(2) $$\Delta E{}_{\mathrm{int}}\left(\zeta \right)=\Delta V{}_{\mathrm{elstat}}\left(\zeta \right)+\Delta E{}_{\mathrm{Pauli}}\left(\zeta \right)+\Delta E{}_{\mathrm{oi}}\left(\zeta \right)$$

Herein, Δ*V*_elstat_(ζ) is the classical electrostatic interaction between the unperturbed charge distributions of the (deformed) reactants and is usually attractive. The Pauli repulsion, Δ*E*_Pauli_(ζ), comprises the destabilizing interaction between occupied closed‐shell orbitals of both fragments due to Pauli's exclusion principle. The orbital interaction energy, Δ*E*_oi_(ζ), accounts, *i. e*., for one‐electron (*e. g*., SOMO–LUMO), electron‐pair (SOMO–SOMO) and three‐electron bonding (*e. g*., SOMO–HOMO), polarization within reactants, and charge transfer between reactants (*e. g*., HOMO–LUMO). A detailed, step‐by‐step, guide on how to perform and interpret the ASM and EDA can be found in Ref. [16c].

The activation strain and energy decomposition analyses were carried out along the intrinsic reaction coordinate (IRC) projected onto a critical geometry parameter, which is, in this study, the shortest of the two newly forming C⋅⋅⋅C bonds between the 1,3‐butadiene and acrylaldehyde. This particular geometry parameter undergoes a well‐defined change during the reaction going from the reactants via the transition state to the cycloadduct and has been shown to be a valid reaction coordinate for studying cycloaddition reactions.[^8^, ^11^]

## Results and Discussion

**Figure 1:**
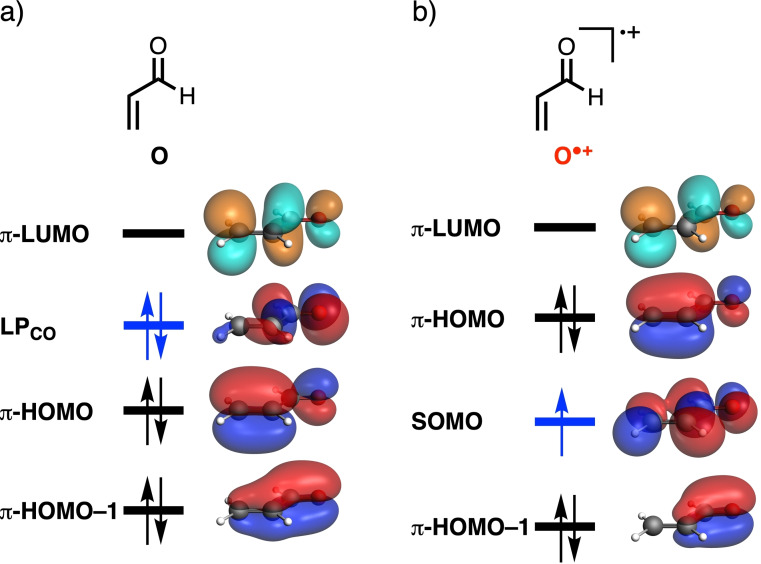
Simplified schematic representation of the molecular orbital diagrams of a) acrylaldehyde **O** and b) the acrylaldehyde radical cation **O^.+^** (isovalue=0.03 Bohr^−3/2^; computed at ZORA‐(U)BP86/TZ2P), where the singly and doubly occupied 3D orbitals are depicted in red and blue, the virtual 3D orbitals are shown in orange and teal, and the molecular orbital energy level from which the electron is removed is highlighted in blue. Note that ionization stabilizes all molecular orbitals.

First, we analyze and compare the molecular orbital diagrams of acrylaldehyde (**O**) and acrylaldehyde radical cation (**O^.+^**) to establish how ionization affects the molecular orbitals of the dienophile that participate in the Diels‐Alder reaction with 1,3‐butadiene (**B**). For clarity, we discuss the nature of the molecular orbitals and their bonding mechanism (*vide infra*) in terms of unoccupied, singly‐occupied, and doubly‐occupied spatial orbitals. Note, however, that all results have been obtained from fully unrestricted DFT calculations involving spin‐polarized orbitals (see Computational Details). For a comprehensive discussion of all details, see the Supporting Information. **O^.+^** is generated by removing an electron from the highest occupied molecular orbital (HOMO) of **O**, which is the LP_CO_ that has the appearance of a carbonyl−oxygen lone pair and has most of its amplitude on that oxygen atom (Figures 1a and 1b). This electron removal generates a hole and a net positive potential which is mostly located on the oxygen atom of the radical‐cation dienophile. The resulting SOMO is pulled down significantly in energy by its one hole and the associated net positive potential (see Figure 1). This net positive potential also pulls all other molecular orbitals of **O^.+^** down in energy. The fact that the generated hole is located largely on the carbonyl oxygen makes this oxygen atom effectively more electronegative and polarizes occupied orbitals of **O^.+^** from the C=C double bond towards the oxygen. Later, we will show that this ionization‐induced polarization plays a key role in both the enhanced reactivity as well as the change from concerted to stepwise in the radical‐cation Diels‐Alder reactions, in line with our previous studies on Lewis acid‐catalyzed Diels‐Alder reactions.^[8]^

**Figure 2:**
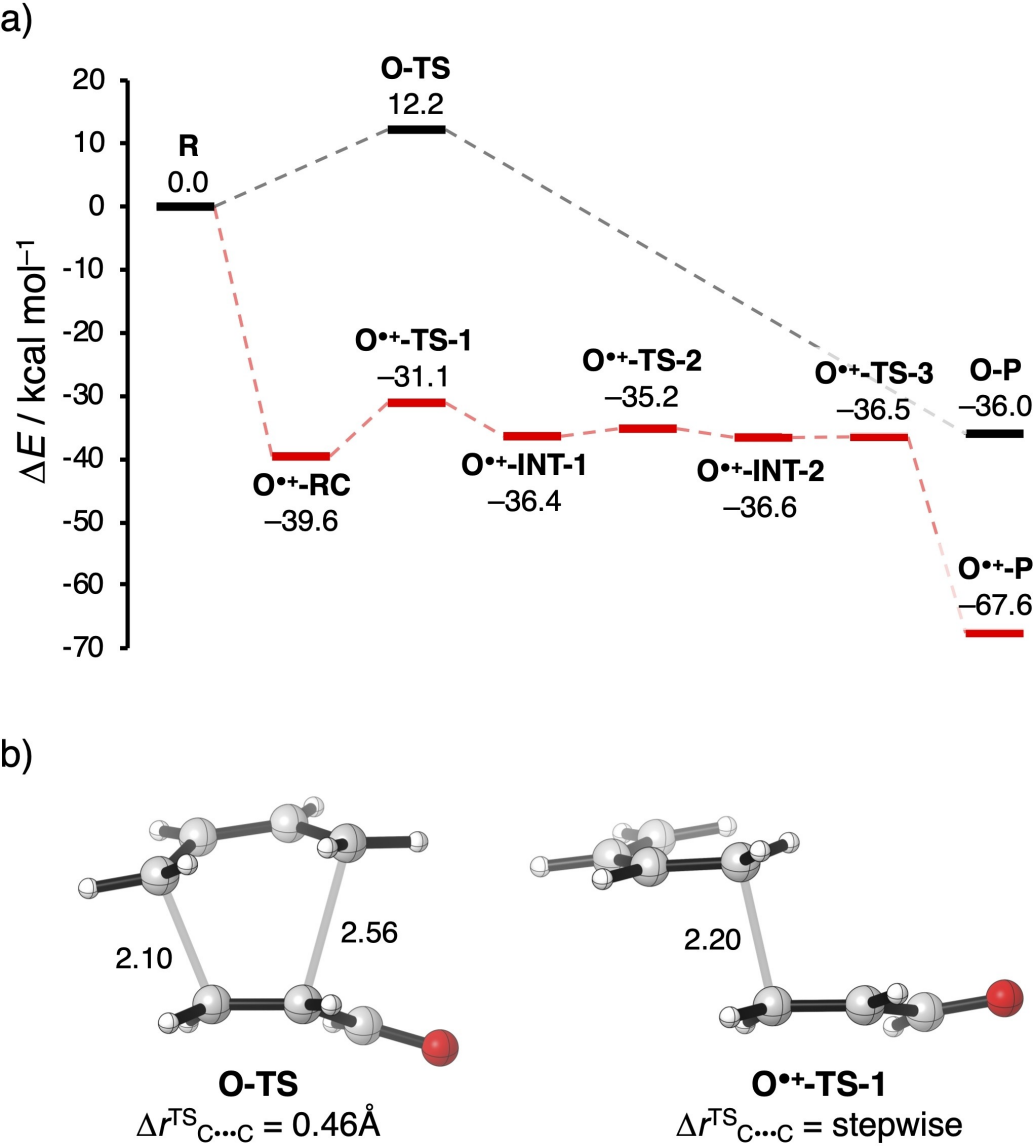
a) Reaction profiles (in kcal mol^−1^) and b) transition state structures (see Figure S1 for all stationary point structures) with newly forming bond distances (in Å) and degree of asynchronicity (Δ*r* ^TS^_C⋅⋅⋅C_) of the neutral and radical‐cation Diels‐Alder reaction between 1,3‐butadiene (**B**) and α,β‐unsaturated dienophile (**X**), computed at ZORA‐(U)BP86/TZ2P.

Next, we examine and compare the neutral and the radical‐cation Diels‐Alder (DA) reactions between butadiene (**B**) and the α,β‐unsaturated dienophile (**X**=**O** and **O^.+^**, respectively). The reaction profiles together with their transition‐state structures of the neutral DA reaction, in which the dienophile is acrylaldehyde (**O**), and the radical‐cation DA reaction, in which the dienophile is acrylaldehyde radical cation (**O^.+^**), are shown in Figure 2 (see Figure S2 for all stationary‐point structures). In line with our previous studies on Lewis acid‐catalyzed Diels‐Alder reactions^[8]^ as well as other studies on radical‐cation Diels‐Alder reactions,^[15]^ we found that Diels‐Alder reactions involving a radical cation as dienophile not only show a significantly enhanced reactivity, but also an increased asynchronicity of the reaction, even to the extent that the reaction pathway becomes stepwise. The neutral DA reaction between **B** and **O** follows a concerted asynchronous reaction mode and hence has a single reaction barrier of 12.2 kcal mol^−1^. The degree of asynchronicity, that is, the difference in distance between the two newly forming C⋅⋅⋅C bonds in the transition state, is 0.46 Å. In contrast, the radical‐cation DA reaction between **B** and **O^.+^** goes via a stepwise mechanism, in which the first step is the formation of the first C−C bond between the terminal carbon of **B** and the β‐carbon of **O^.+^**, which has a relative energy of −31.1 kcal mol^−1^ with respect to the separate reactants (8.5 kcal mol^−1^ with respect to the preceding reactant complex **O^.+^‐RC**) and results in an intermediate where the two original reactants are in an *anti*‐conformation (**O^.+^‐INT‐1**). The second step is the rotation around the newly formed C−C bond, yielding an intermediate where the two (original) reactants are in a *gauche*‐conformation (**O^.+^‐INT‐2**). The final step is the formation of the second C−C bond, *i. e*., ring closure, yielding the cycloadduct **O^.+^‐P**.

Next, we examine the physical factors leading to the enhanced reactivity of the radical‐cation compared to the neutral Diels‐Alder reaction, by applying the activation strain model (ASM) of reactivity,^[16]^ where the interacting fragments are the 1,3‐butadiene (**B**) and α,β‐unsaturated dienophile (**X**). Here, we show the results of this analysis on consistent, transition state‐like geometries for the DA reactions with both **O** and **O^.+^** (Table 1), which are acquired via an IRC calculation and have a C_**B**_⋅⋅⋅C_**Xβ**_ distance of 2.203 Å between the terminal carbon of **B** and the β‐carbon of **X** (**X**=**O** and **O^.+^**), that is, they are both at the same point along the reaction coordinate (see Figure S3 for the analysis along the entire reaction coordinate). Performing this analysis at a consistent point along the reaction coordinate (near both transition‐state structures), rather than the transition state alone, ensures that the results are not skewed by the position of the transition state along the reaction coordinate, *i. e*., by the differences in distances between the reactants.[^16^, ^24^] We compare and analyze the first reaction step, which is rate‐determining for both reactions, **B**+**O** and **B**+**O^.+^** (and which is the only reaction step for the neutral reaction system). The trend in total energies at the consistent geometries (ΔΔE*=−42.5 kcal mol^−1^ for **O^.+^** relative to **O**) is in line with the observed reactivity trend of the actual reaction barriers (ΔΔ*E* ^≠^=−43.3 kcal mol^−1^), that is, the radical‐cation DA reaction goes with a lower, less destabilizing total energy compared to the neutral analog (Table 1). The enhanced reactivity of the radical‐cation DA reaction can, in turn, be traced back to both a less destabilizing strain energy (ΔΔ*E*_strain_=−5.1 kcal mol^−1^) and a significantly more stabilizing interaction energy (ΔΔ*E*_int_=−37.4 kcal mol^−1^).

**Table 1:** Activation strain and energy decomposition analyses (in kcal mol^−1^) of the neutral and radical‐cation Diels‐Alder reactions between **B** and **X**.^[a,b,c]^

**X**	ΔE*	Δ*E*_strain_	Δ*E*_int_	Δ*V*_elstat_	Δ*E*_Pauli_	Δ*E*_oi_
**O**	11.4	14.6	−3.2	−32.7	70.8	−41.3
**O^.+^**	−31.1	9.5	−40.6	−23.8	52.0	−68.8

[a] Where the interacting fragments are the 1,3‐butadiene (**B**) and α,β‐unsaturated dienophile (**X**). [b] Analyses at consistent transition state‐like geometries with a C_**B**_⋅⋅⋅C_**Xβ**_ distance of 2.203 Å between **B** and the β‐carbon of **X** at ZORA‐(U)BP86/TZ2P. [c] See Figure S3 for the complete potential energy surface.

The differences in strain energy are directly related to the differences in reaction mode.^[9]^ The neutral DA reaction between **B** and **O** is concerted asynchronous, which means that all carbon atoms of **B** and **O**, involved in the formation of the two new C⋅⋅⋅C bonds during the DA reaction, are pyramidalizing. In contrast, the radical‐cation DA reaction is stepwise and, therefore, experiences, during the first reaction step, less geometrical deformation and, ultimately, less destabilizing strain energy, because only the β‐carbon of **O^.+^** and one of the terminal carbon atoms of **B** are pyramidalizing upon forming the first C_**B**_−C_**Xβ**_ bond.

To understand why the interaction energy becomes more stabilizing when going from a neutral to a radical‐cation DA reaction, we have carried out our canonical energy decomposition analysis (EDA).^[19]^ We find that both a reduced destabilizing Pauli repulsion (ΔΔ*E*_Pauli_=−18.8 kcal mol^−1^) and a more stabilizing orbital interactions (ΔΔ*E*_oi_=−27.5 kcal mol^−1^) contribute to the more stabilizing interaction energy for the radical‐cation compared to the neutral DA reaction. The electrostatic interaction, on the other hand, shows an opposite trend (ΔΔ*V*_elstat_=+8.9 kcal mol^−1^, *i. e*., the radical‐cation reaction goes with a less stabilizing electrostatic interaction) and is, therefore, not responsible for the observed rate enhancement.

**Figure 3:**
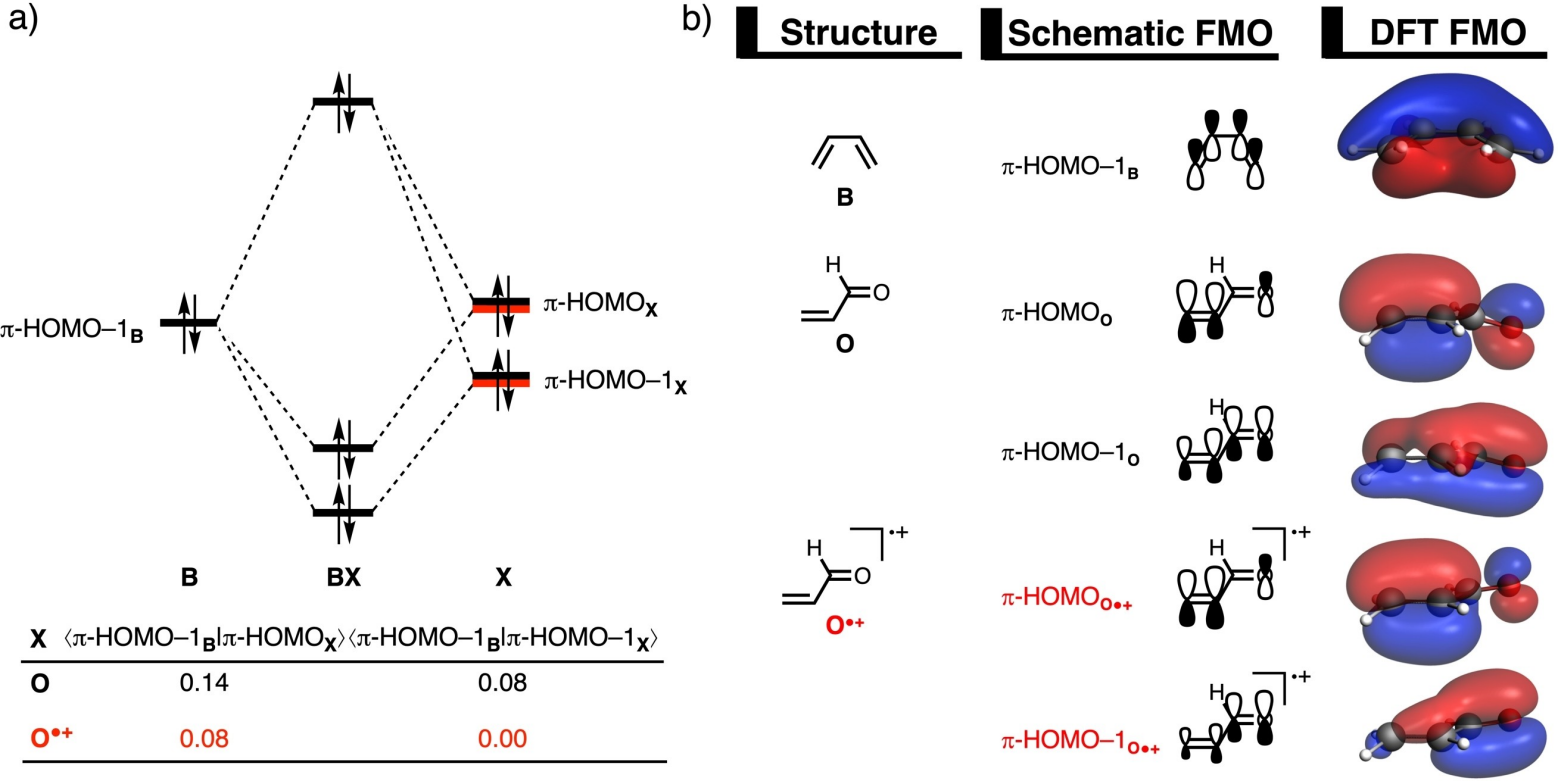
a) Schematic molecular orbital diagram and the most significant closed‐shell‐closed‐shell orbital overlaps of the Diels‐Alder reaction between diene **B** and dienophile **X**; and b) key occupied orbitals (isovalue=0.03 Bohr^−3/2^) of **B**, **O**, and **O^.+^**, computed at consistent TS‐like geometries **BX**, *i. e*., new C_**B**_⋅⋅⋅C_**Xβ**_ bond distance is 2.203 Å for **X**=**O** and **O^.+^** (see text) at ZORA‐(U)BP86/TZ2P.

The origin of the less destabilizing Pauli repulsion for the radical‐cation Diels‐Alder reaction was further investigated by performing a Kohn‐Sham molecular orbital (KS‐MO)[^17^, ^18^] analysis (Figure 3). The key occupied molecular orbitals of **B**, **O**, and **O^.+^** that engage in the repulsive closed–shell‐closed‐shell orbital interaction, were quantified again at the earlier introduced consistent, transition state‐like geometries, in which the newly forming C_**B**_⋅⋅⋅C_**Xβ**_ bond between **B** and **X** is at a distance of 2.203 Å (**X**=**O** and **O^.+^**). The most important orbital of **B** involved in this repulsive interaction is the π‐HOMO−1_**B**_, where all carbon 2*p_z_* atomic orbitals (AOs) are in phase. The participating occupied orbitals of **O** and **O^.+^** are the π‐HOMO and π‐HOMO−1, where the former has one nodal plane in the π‐orbital system between the carbonyl and olefinic moieties of the dienophile and for the latter all 2*p_z_* AOs of the carbonyl and olefinic moieties of the dienophile are in‐phase. The orbital overlap between the π‐HOMO−1_**B**_ and the occupied orbitals of the dienophile is larger and more destabilizing for the neutral DA reaction involving **O** (*S*=0.14 and 0.08) and smaller and less destabilizing for the radical‐cation DA reaction with **O^.+^** (*S*=0.08 and 0.00) (Figure 3a). The differences in the repulsive orbital overlap between **O** and **O^.+^** are a direct consequence of their difference in reaction mode (*vide infra*). Along the concerted asynchronous reaction mode of the neutral DA reaction, there is repulsive orbital overlap at both the α‐ and β‐carbon side of the dienophile. The stepwise reaction mode of the radical‐cation DA reaction, however, precludes, due to the evasive orientation of the reactants (see Figure 2b, right), any repulsive orbital overlap at the α‐carbon of **O^.+^**, manifesting in a reduction of repulsive orbital overlap between **B** and **O^.+^**.^[32]^ Besides following a different reaction mode, the occupied π‐orbitals of **O^.+^** has a smaller orbital amplitude on the β‐carbon compared to **O**, which, in turn, also leads to an additional reduction of repulsive orbital overlap. Notably, we have found these exact phenomena behind the rate acceleration of radical‐cation DA reactions also for Lewis acid‐catalyzed Diels‐Alder reactions.^[8]^ All this demonstrates that the concept of *Pauli‐lowering catalysis* is a more widely occurring phenomenon and not only limited to Lewis acid‐catalyzed Diels‐Alder reactions.

**Figure 4:**
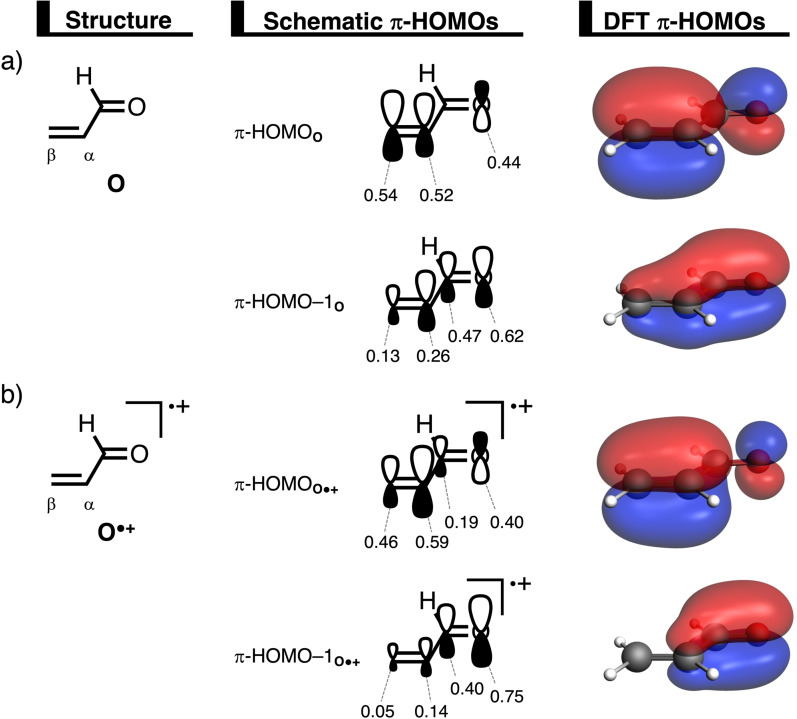
Key occupied π‐HOMOs (isovalue=0.03 Bohr^3/2^) computed at the equilibrium structures of a) acrylaldehyde (**O**) and b) acrylaldehyde radical cation (**O^.+^**), where the MO‐coefficients of the carbon and oxygen 2*p*_z_ atomic orbital, contributing to the occupied orbitals, are shown in the schematic π‐HOMOs, computed at ZORA‐(U)BP86/TZ2P.

Thus, the difference in reaction mode from concerted to stepwise is a crucial factor for the rate enhancement of the radical‐cation compared to the neutral DA reaction, as it reduces both the destabilizing activation strain as well as the repulsive orbital overlap and hence the Pauli repulsion. Where does this change from a concerted to a stepwise mechanism come from? In our previous work,^[9]^ we have shown that the reaction mode of a Diels‐Alder reaction is determined by the asymmetry in the MO‐coefficients of the 2*p*_z_ atomic orbitals (AOs) on the α‐ and β‐carbon atoms of the occupied π‐orbitals of the dienophile. Figure 4 displays the MO‐coefficients of the 2*p*_z_ AOs on the α‐ and β‐carbon atoms of the π‐HOMO and π‐HOMO−1 of **O** and **O^.+^**. The π‐HOMO−1_**O**_ has a smaller orbital amplitude on the β‐carbon compared to the α‐carbon, in terms of 2*p*_z_ AO coefficients, 0.13 on C^β^ *versus* 0.26 on C^α^, causing a larger, more repulsive orbital overlap between **B** and the α‐carbon of **O**. This asymmetry introduces a bias towards forming the C_**B**_⋅⋅⋅C_**Oβ**_ bond ahead of the C_**B**_⋅⋅⋅C_**Oα**_ bond, to reduce this repulsive orbital overlap, and hence gives rise to the observed concerted asynchronous reaction mode for the neutral DA reaction between **B** and **O**. Note that the π‐HOMO_**O**_ slightly counteracts the asynchronicity set by the π‐HOMO−1_**O**_, because the former orbital has a larger orbital amplitude on the β‐carbon compared to the α‐carbon, in terms of 2*p*_z_ AO coefficients, 0.54 on C^β^ *versus* 0.52 on C^α^. As previously discussed, ionizing the dienophile polarizes the π‐HOMO_**O⋅+**_ and π‐HOMO−1_**O⋅+**_ towards the electronegative carbonyl oxygen, thereby amplifying the asymmetry of these orbitals on the α‐ and β‐carbon atoms, in terms of 2*p*_z_ AO coefficients, that is, π‐HOMO_**O⋅+**_: 0.46 on C^β^ *versus* 0.59 on C^α^; π‐HOMO−1_**O⋅+**_: 0.05 on C^β^ *versus* 0.14 on C^α^ (Figure 4b). Consequently, the highly repulsive orbital overlap between **B** and the α‐carbon of **O^.+^**, compared to the β‐carbon of **O^.+^**, increases the bias towards forming the C_**B**_⋅⋅⋅C_**O⋅+**β_ bond ahead of the C_**B**_⋅⋅⋅C_**O⋅+**α_ bond to such an extent that it affects the orientation of the approaching reactants, making the radical‐cation DA reaction between **B** and **O^.+^** follow a stepwise reaction mode.

**Figure 5:**
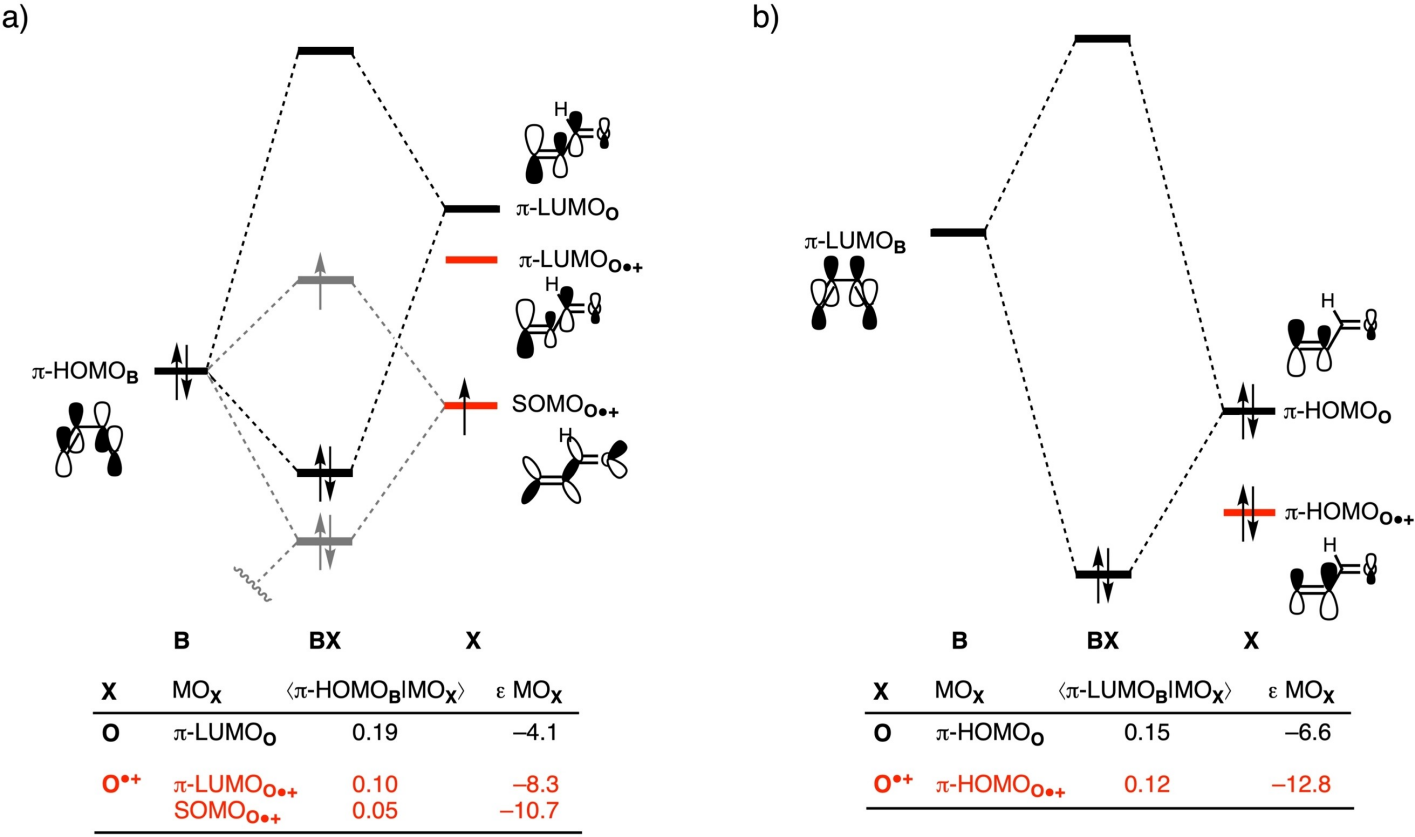
Schematic molecular orbital diagrams with key orbital interactions for a) the normal electron demand (NED) π‐HOMO_**B**_–MO_**X**_ interaction, where the additional π‐HOMO_**B**_–SOMO_**O⋅+**_ interaction is shown in grey; and b) inverse electron demand (IED) π‐LUMO_**B**_–MO_**X**_ of the neutral and radical‐cation Diels‐Alder reaction between diene **B** and dienophile **X**, computed at consistent TS‐like geometries **BX**, *i. e*., new C_**B**_⋅⋅⋅C_**Xβ**_ bond distance is 2.203 Å for **X**=**O** and **O^.+^** (see text) at ZORA‐(U)BP86/TZ2P.

Finally, we address why the radical‐cation DA reaction also benefits from more stabilizing orbital interactions compared to the neutral reaction, in addition to having less Pauli repulsion and less activation strain. Ionization of the dienophile strengthens the normal electron demand (NED), but, simultaneously, weakens the inverse electron demand (IED) orbital interactions. As discussed above, all molecular orbitals of the dienophile are stabilized when an electron is removed from the dienophile. Therefore, the π‐HOMO_**B**_–π‐LUMO_**O⋅+**_ energy gap is reduced, which makes the NED interaction more favorable for the radical‐cation compared to the neutral DA reaction (Figure 5a). Additionally, the π‐LUMO_**B**_–π‐HOMO_**O⋅+**_ energy gap is enlarged and hence the IED interaction becomes less stabilizing for the radical‐cation compared to the neutral DA reaction (Figure 5b). These two effects mutually approximately cancel and have little effect on the overall strength of the combined orbital interactions, similar to situations we previously found in the case of LA catalysis.[^8^, ^9^]

The above raises the question of what does cause the enhanced orbital interactions for the radical‐cation reaction. It is, in fact, the low‐energy SOMO_**O⋅+**_, which is able to accept electrons from the π‐HOMO_**B**_, *i. e*., it engages in a three‐electron bonding interaction, that, ultimately, gives rise to the more stabilizing orbital interactions for the radical‐cation compared to the neutral DA reaction. By performing a Kohn‐Sham molecular orbital (KS‐MO)[^17^, ^18^] analysis on consistent, transition state‐like geometries with a C_**B**_⋅⋅⋅C_**Xβ**_ distance of 2.203 Å between **B** and **X** (**X**=**O** and **O^.+^**),[^17^, ^18^] we find that the net positive potential of the radical cation stabilizes all orbitals on **O^.+^** and in particular the SOMO_**O⋅+**_ that originally held the electron that has been removed (*vide supra*: see Figures 1 and 5a; see also Figure S7 for comprehensive molecular orbital diagrams based on the spin‐orbitals). Thus, the SOMO_**O⋅+**_ enters into a three‐electron bonding interaction with the π‐HOMO_**B**_ with which it has a moderate overlap of 0.05 and from which it accepts 0.7 electrons. Such a stabilizing three‐electron bond is not possible in the neutral, closed‐shell DA reaction and, therefore, provides an additional stabilizing contribution to the orbital interactions in the radical‐cation *versus* the neutral DA reaction.

## Conclusion

Ionization of the dienophile accelerates the Diels‐Alder reaction between 1,3‐butadiene (**B**) and acrylaldehyde (**O**). It changes the reaction mechanism from concerted asynchronous, for the neutral Diels‐Alder reaction, to stepwise, for the radical‐cation Diels‐Alder reaction. These findings emerge from our detailed quantum chemical analyses based on the activation strain model and quantitative molecular orbital theory as contained in Kohn‐Sham density functional theory (DFT).

Ionization of the dienophile occurs from the LP_CO_, a molecular orbital with significant lone‐pair character on the carbonyl oxygen which generates a hole and net positive potential that lends the oxygen atom a higher effective electronegativity. The latter induces a polarization of all occupied π‐orbitals away from the C=C double bond towards the oxygen atom. Importantly, it amplifies the asymmetry of the dienophile‘s occupied π‐orbitals such that their amplitude on the α‐carbon becomes significantly more prominent than on the β‐carbon. This polarization has two significant consequences for inducing a kinetic rate enhancement: i) it results in a loss of destabilizing Pauli repulsion between the occupied π‐orbitals of the diene and dienophile; and ii) it alters the reaction mode from concerted asynchronous to stepwise, which, due to a more evasive mutual orientation of the reactants, further reduces the Pauli repulsion and activation strain.

Ionization also promotes the stabilizing orbital interactions between the diene and dienophile by introducing an additional three‐electron bonding interaction between the SOMO of the radical‐cation dienophile and the HOMO of the diene. In the neutral Diels‐Alder reaction, the same pair of orbitals are involved in a closed‐shell–closed‐shell Pauli repulsive interaction. The normal electron demand HOMO_diene_–LUMO_dienophile_ interaction also becomes more stabilizing for the radical‐cation Diels‐Alder reaction, but this enhancement is offset by the significant weakening of the inverse electron demand LUMO_diene_–HOMO_dienophile_ interaction. Our findings reveal the similarities and differences behind the Diels‐Alder rate enhancement through ionization *versus* Lewis acid catalysis.

## Conflict of interest

The authors declare no conflict of interest.

1

## Supporting information

As a service to our authors and readers, this journal provides supporting information supplied by the authors. Such materials are peer reviewed and may be re‐organized for online delivery, but are not copy‐edited or typeset. Technical support issues arising from supporting information (other than missing files) should be addressed to the authors.

Supporting Information

## Data Availability

The data that support the findings of this study are available in the supplementary material of this article.
