# Supplementary material for: How Ionization Catalyzes Diels‐Alder Reactions
Source: Chemistry. 2022 May 13;28(40):e202200987. doi: 10.1002/chem.202200987 (PMC9400981; doi:10.1002/chem.202200987)
Supplement: Supplementary file 1 — Supporting Information [file CHEM-28-0-s001.pdf]

Chemistry–A European Journal

Supporting Information

How Ionization Catalyzes Diels-Alder Reactions

Pascal Vermeeren, Trevor A. Hamlin,* and F. Matthias Bickelhaupt*

Contents

Table S1. Expectation value $\langle S^2 \rangle$ for all stationary points of the radical-cation Diels-Alder reaction, computed at ZORA-(U)BP86/TZ2P level.

Figure S1. Key molecular orbitals of a) acrylaldehyde **O** and b) the acrylaldehyde radical cation $\mathbf{O}^{\bullet+}$ (orbital energies in eV displayed in red for α- and β-spin orbitals). The molecular orbital from which the electron is removed is highlighted in blue.

Figure S2. Structures of stationary points of the neutral and radical-cation Diels-Alder reaction of 1,3-butadiene (**B**) with the α,β-unsaturated dienophile (**X**), computed at ZORA-BP86/TZ2P.

Figure S3. a) Activation strain analyses and b) energy decomposition analyses of the neutral and radical-cation Diels-Alder reactions between **B** and **X**, along the IRC from reactants to transition state (indicated with a dot), projected on the shorter newly forming $C_{\mathbf{B}}\bullet\bullet\bullet C_{\mathbf{X}\beta}$ bond between **B** and **X**, computed at ZORA-(U)BP86/TZ2P.

Table S2. Activation strain and energy decomposition analyses (in kcal mol^{-1}) of the neutral and radical-cation Diels-Alder reactions between **B** and **X**, following the reaction pathway of the neutral Diels-Alder reaction.

Figure S4. a) Activation strain analyses and b) energy decomposition analyses of the neutral and radical-cation Diels-Alder reactions between **B** and **X**, following the reaction pathway of the neutral Diels-Alder reaction, along the IRC from reactants to transition state (indicated with a dot), projected on the shorter newly forming $C_{\mathbf{B}}\bullet\bullet\bullet C_{\mathbf{X}\beta}$ bond between **B** and **X**, computed at ZORA-(U)BP86/TZ2P.

Figure S5. a) Schematic molecular orbital diagram and the most significant closed-shell–closed-shell orbital overlaps of the Diels-Alder reaction between diene **B** and dienophile **X**, following the reaction pathway of the neutral Diels-Alder reaction; and b) key occupied orbitals (isovalue = 0.03 Bohr$^{-3/2}$) of **B**, **O**, and $\mathbf{O}^{\bullet+}$, computed at consistent TS-like geometries **BX**, *i.e.*, new $C_{\mathbf{B}}\bullet\bullet\bullet C_{\mathbf{X}\beta}$ bond distance is 2.107 Å for **X** = **O** and $\mathbf{O}^{\bullet+}$ (see text), computed at ZORA-(U)BP86/TZ2P.

Figure S6. Activation strain analyses: (a) total energy, (b) strain energy, and (c) interaction energy; and energy decomposition analyses: (d) Pauli repulsion, (e) electrostatic interaction, and (f) orbital interactions, of the artificially constraint synchronous, asynchronous, and stepwise Diels-Alder reaction modes (rxn mode) between **B** and $\mathbf{O}^{\bullet+}$, where the transition states are indicated with a dot and the energy values are projected onto the shorter newly forming a $C_{\mathbf{B}}\bullet\bullet\bullet C_{\beta}$ bond, computed at ZORA-(U)BP86/TZ2P.

Figure S7. Schematic molecular orbital diagrams with key orbital interactions for a) the neutral Diels-Alder reaction between **B** and **O** and b) the radical-cation Diels-Alder reaction between **B** and $\mathbf{O}^{\bullet+}$, computed at consistent TS-like geometries **BX**, *i.e.*, new $C_{\mathbf{B}}\bullet\bullet\bullet C_{\mathbf{X}\beta}$ bond distance is 2.203 Å for **X** = **O** and $\mathbf{O}^{\bullet+}$ (see text) at ZORA-(U)BP86/TZ2P, where the three-electron bonding interaction of the radical-cation Diels-Alder reaction is shown in red, the fragment orbital energies in the presence of the other reactant (black, in eV), gross Mulliken populations of fragment orbitals (blue, in electrons).

Figure S8. Representation of the overlapping π-HOMO$_{\mathbf{B}}$ and SUMO$_{\mathbf{O}^{\bullet+}}$ (isovalue = 0.03 Bohr$^{-3/2}$) at consistent TS-like geometries **BX** (*i.e.*, new $C_{\mathbf{B}}$•••$C_{\mathbf{X}\beta}$ bond distance is 2.203 Å for **X** = **O** and **O**$^{\bullet+}$; see text) at ZORA-(U)BP86/TZ2P.

Table S3. Cartesian coordinates (in Å), ADF total energies (in kcal mol^{-1}), and number of imaginary frequencies of all stationary points, computed at ZORA-(U)BP86/TZ2P.

Table S1. Expectation value $\langle S^2 \rangle$ for all stationary points of the radical-cation Diels-Alder reaction, computed at ZORA-(U)BP86/TZ2P level.[a]

Stationary Point	$\langle S^2 \rangle$
**O**$^{\bullet+}$	0.75579
**2-RC**	0.75589
**2-TS-1**	0.76469
**2-INT-1**	0.76939
**2-TS-2**	0.76624
**2-INT-2**	0.76211
**2-TS-3**	0.76173
**2-P**	0.75248

[a] The $\langle S^2 \rangle$ values in the ADF algorithm are calculated via the formula implemented by Bulo *et al.* (See Ref. 29 of R. E. Bulo, A. W. Ehlers, S. Grimme, K. Lammertsma, *J. Am. Chem. Soc.* **2002**, *124*, 13903).

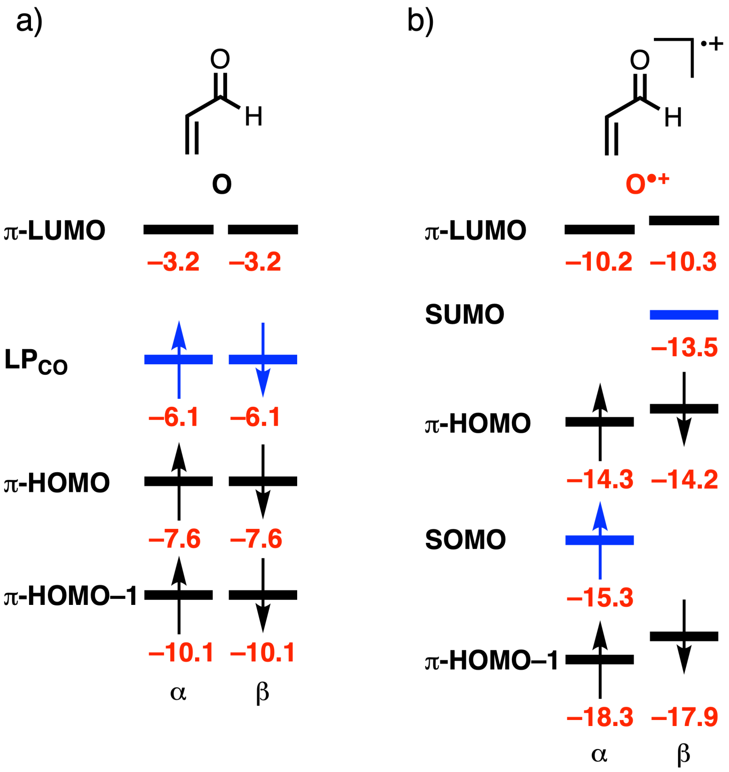

Figure S1. Key molecular orbitals of a) acrylaldehyde **O** and b) the acrylaldehyde radical cation $\mathbf{O}^{\bullet+}$ (orbital energies in eV displayed in red for α- and β-spin orbitals). The molecular orbital from which the electron is removed is highlighted in blue.

Here, we discuss the effect of ionization on the molecular orbitals of the dienophile (**X**) that participate in the Diels-Alder reaction with 1,3-butadiene in terms of their spin-polarized orbitals, that is, unoccupied (LUMO), singly-unoccupied (SUMO), singly-occupied (SOMO), and doubly-occupied (HOMO) α- and β-spin orbitals. The acrylaldehyde radical cation ($\mathbf{O}^{\bullet+}$) is generated by removing an electron from the highest occupied molecular orbital (HOMO) of acrylaldehyde (**O**), which is the LP_{CO} that has the appearance of a carbonyl-oxygen lone pair and has most of its amplitude on that oxygen atom (Figure 1a and 1b). As a result, all orbitals are stabilized due to the reduced electron–electron Coulomb repulsion. The spin-orbitals carrying the electrons with the spin that is in excess (spin up, *i.e.*, α-spin, in Figure S1) are lowered in energy somewhat more due to favorable exchange effects. The LP_{CO}, from which ionization occurs, undergoes the most significant splitting in energy into a substantially stabilized singly occupied molecular orbital (SOMO: –15.3 eV), which has been relieved from Coulomb repulsion with its partner electron that was ionized from the LP_{CO}, and a less stabilized singly unoccupied molecular orbital (SUMO: –13.5 eV), which still experiences virtual repulsion with the electron in the SOMO. Interestingly, this circumstance makes the

stabilization of the SOMO is larger than that of the π-HOMO, causing the former orbital to be energetically below the π-HOMO, and hence $\mathbf{O}^{\bullet+}$ can be labeled as a SOMO/HOMO inversion (SHI) radical.[1]

Besides altering the stability of the molecular orbitals, radical cation formation also has a profound effect on the spatial distribution of the molecular orbitals of $\mathbf{O}^{\bullet+}$ (Figure 1c and 1d). As prior mentioned, ionization leads to the removal of an electron out of the LP_{CO} and generates a hole and a net positive potential which is mostly located on the oxygen atom, making it effectively more electronegative. As a result, all occupied orbitals of $\mathbf{O}^{\bullet+}$ are polarized towards this more electronegative oxygen atom. This effect is most pronounced for the π-HOMO–1, where all carbon and oxygen $2p_z$ atomic orbitals (AOs) are in-phase, which for **O** has orbital amplitude on the β-carbon whereas for $\mathbf{O}^{\bullet+}$ this orbital amplitude on the β-carbon is diminished. Notably, we found that Lewis acids and iminium ions coordinated to the dienophile have the same effect on the shape of molecular π-orbitals by partly abstracting an electron from the dienophile, due to a strong donor–acceptor interaction, and hence polarizing the occupied π-orbitals towards the carbonyl oxygen.[2]

---

[1] L. Abella, J. Crassous, L. Favereau, J. Autschbach, *Chem. Mater.* **2021**, *33*, 3678.

[2] a) P. Vermeeren, T. A. Hamlin, I. Fernández, F. M. Bickelhaupt, *Angew. Chem. Int. Ed.* **2020**, *59*, 6201; *Angew. Chem.* **2020**, *132*, 6260; b) P. Vermeeren, T. A. Hamlin, I. Fernández, F. M. Bickelhaupt, *Chem. Sci.* **2020**, *11*, 8105; c) P. Vermeeren, T. A. Hamlin, F. M. Bickelhaupt, I. Fernández, *Chem. Eur. J.* **2021**, *27*, 5180; d) P. Vermeeren, M. Dalla Tiezza, M. van Dongen, I. Fernández, F. M. Bickelhaupt, T. A. Hamlin, *Chem. Eur. J.* **2021**, *27*, 10620; e) T. A. Hamlin, I. Fernández, F. M. Bickelhaupt, *Angew. Chem. Int. Ed.* **2019**, *58*, 8922; *Angew. Chem.* **2019**, *131*, 9015.

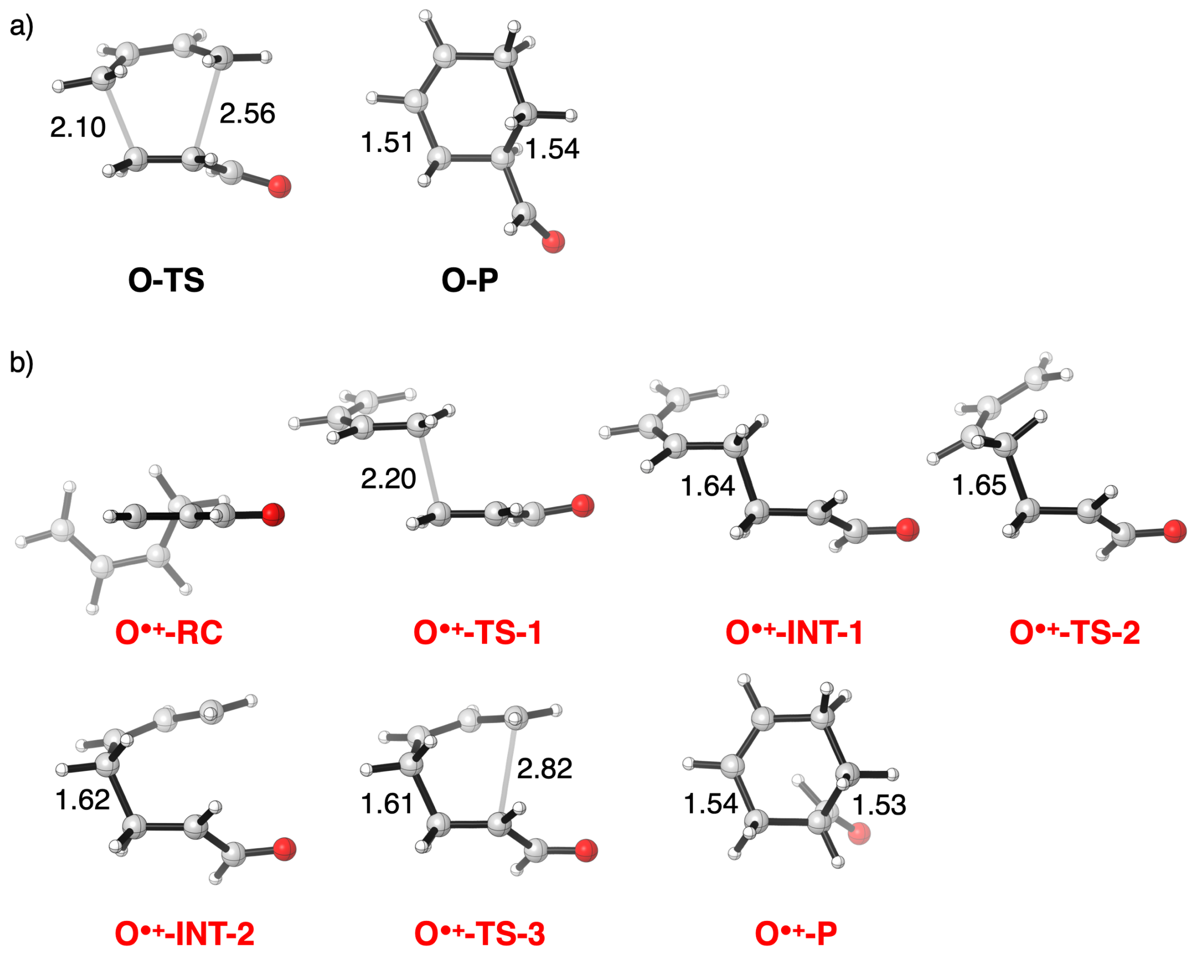

Figure S2. Structures of stationary points of the neutral and radical-cation Diels-Alder reaction of 1,3-butadiene (**B**) with the α,β-unsaturated dienophile (**X**), computed at ZORA-(U)BP86/TZ2P.

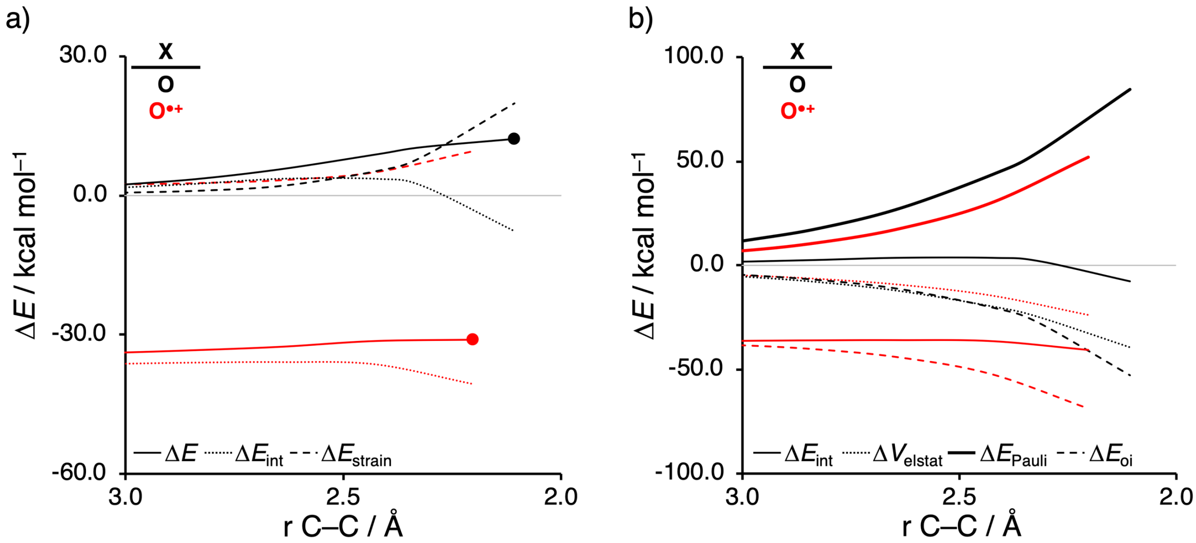

Figure S3. a) Activation strain analyses and b) energy decomposition analyses of the neutral and radical-cation Diels-Alder reactions between **B** and **X**, along the IRC from reactants to transition state (indicated with a dot), projected on the shorter newly forming C_B•••$C_{X\beta}$ bond between **B** and **X**, computed at ZORA-(U)BP86/TZ2P.

Table S2. Activation strain and energy decomposition analyses (in kcal mol^{-1}) of the neutral and radical-cation Diels-Alder reactions between **B** and **X**, following the reaction pathway of the neutral Diels-Alder reaction.[a]

X	ΔE^*	ΔE_{strain}	ΔE_{int}	ΔV_{elstat}	ΔE_{Pauli}	ΔE_{oi}
**O**	12.2	19.9	–7.7	–39.4	84.5	–52.8
**O**$^{\bullet+}$	–22.6	20.8	–43.4	–35.5	74.1	–82.0

[a] Analyses at consistent transition state-like geometries with a $C_B \cdots C_{X\beta}$ distance of 2.107 Å between **B** and the β-carbon of **X** at ZORA-(U)BP86/TZ2P.

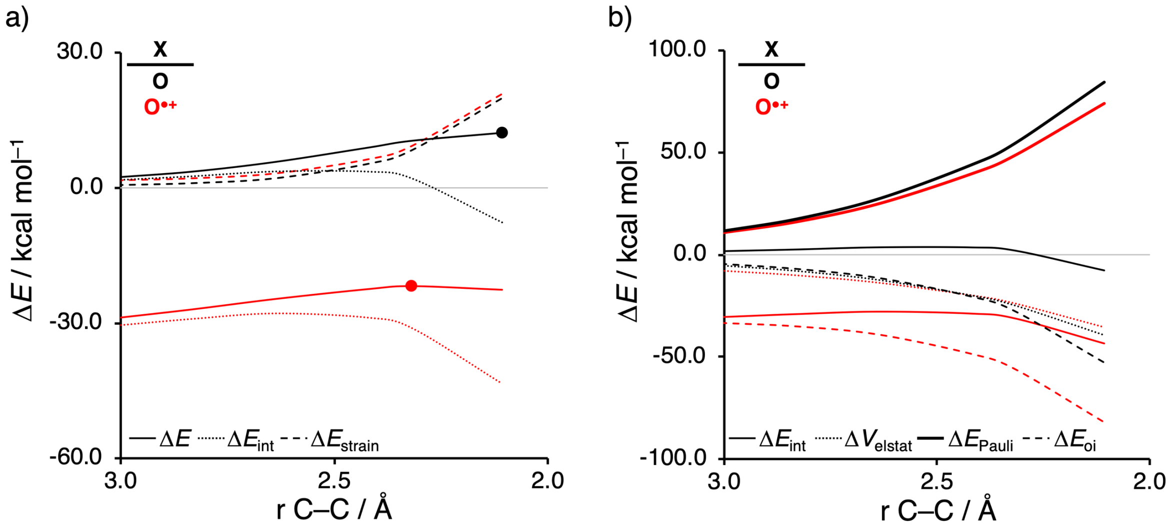

Figure S4. a) Activation strain analyses and b) energy decomposition analyses of the neutral and radical-cation Diels-Alder reactions between **B** and **X**, following the reaction pathway of the neutral Diels-Alder reaction, along the IRC from reactants to transition state (indicated with a dot), projected on the shorter newly forming $C_B \cdots C_{X\beta}$ bond between **B** and **X**, computed at ZORA-(U)BP86/TZ2P.

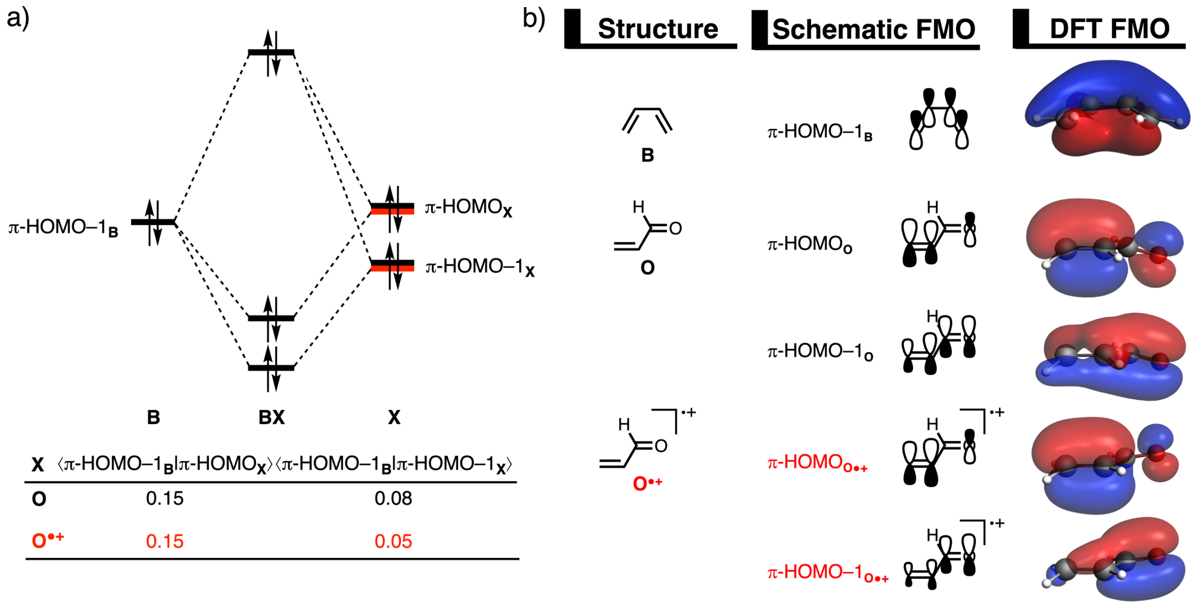

Figure S5. a) Schematic molecular orbital diagram and the most significant closed-shell–closed-shell orbital overlaps of the Diels-Alder reaction between diene **B** and dienophile **X**, following the reaction pathway of the neutral Diels-Alder reaction; and b) key occupied orbitals (isovalue = 0.03 Bohr$^{-3/2}$) of **B**, **O**, and **O$^{\bullet+}$**, computed at consistent TS-like geometries **BX**, *i.e.*, new $C_{\mathbf{B}}$•••$C_{\mathbf{X}\beta}$ bond distance is 2.107 Å for **X** = **O** and **O$^{\bullet+}$** (see text), computed at ZORA-(U)BP86/TZ2P.

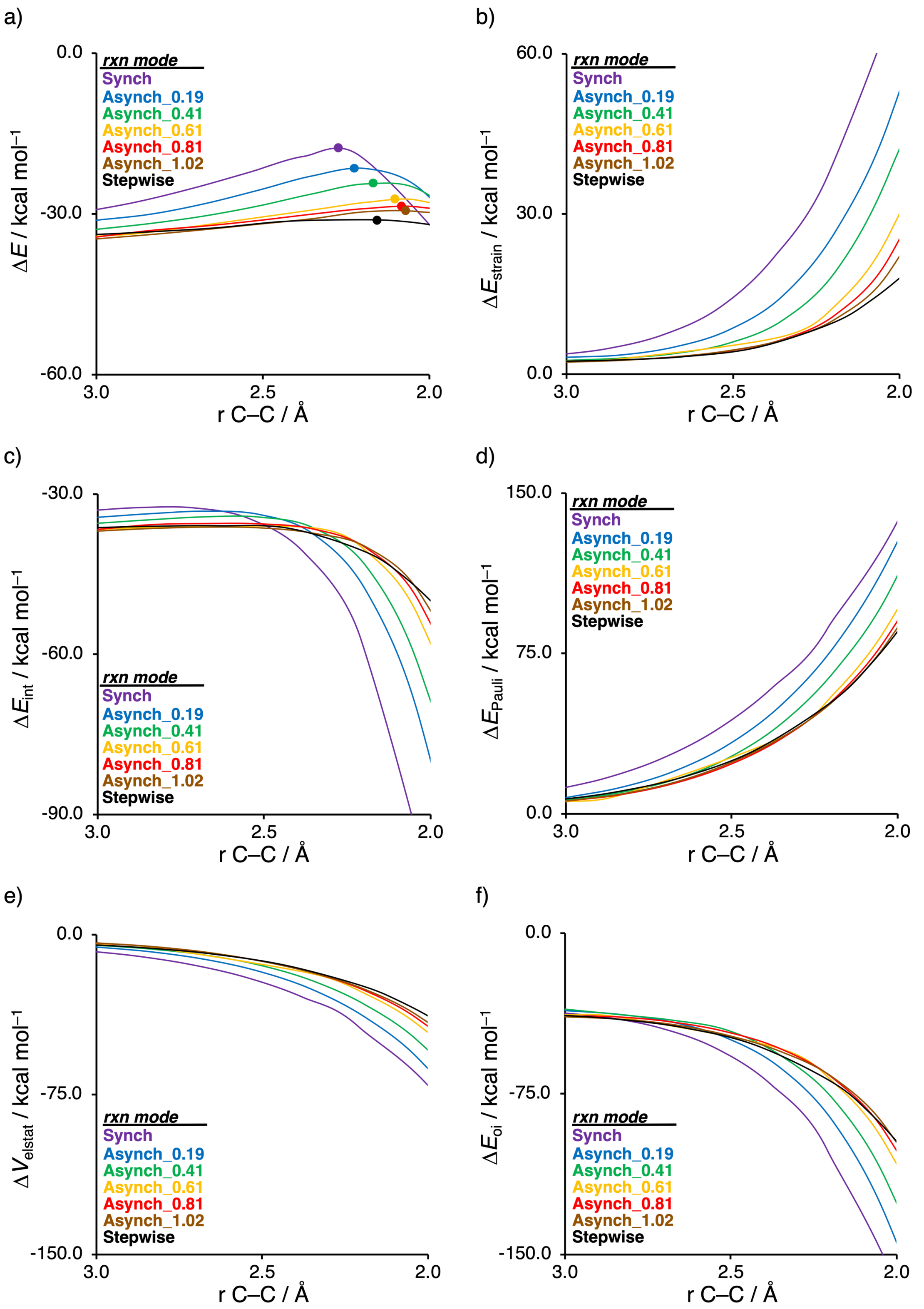

Figure S6. Activation strain analyses: (a) total energy, (b) strain energy, and (c) interaction energy; and energy decomposition analyses: (d) Pauli repulsion, (e) electrostatic interaction, and (f) orbital interactions, of the artificially constraint synchronous, asynchronous, and stepwise Diels-Alder reaction modes (rxn mode) between **B** and **O**$^{\bullet+}$, where the transition states are indicated with a dot and the energy values are projected onto the shorter newly forming a $C_{\mathbf{B}}$•••C_{β} bond, computed at ZORA-(U)BP86/TZ2P.

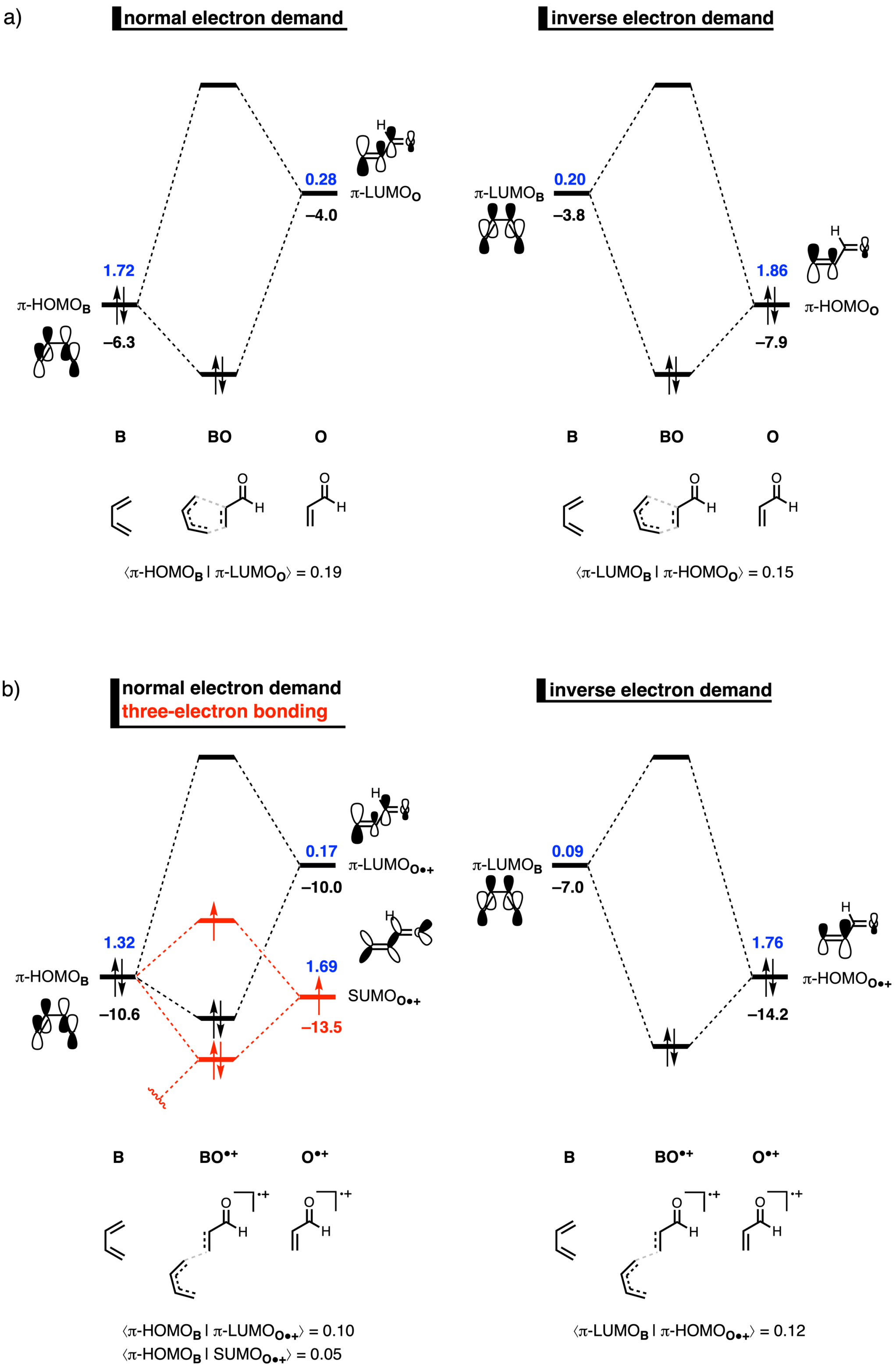

Figure S7. Schematic molecular orbital diagrams with key orbital interactions for a) the neutral Diels-Alder reaction between **B** and **O** and b) the radical-cation Diels-Alder reaction between **B** and $\mathbf{O}^{\bullet+}$, computed at consistent TS-like geometries **BX**, *i.e.*, new $C_{\mathbf{B}}\bullet\bullet\bullet C_{\mathbf{X}\beta}$ bond distance is 2.203 Å for **X** = **O** and $\mathbf{O}^{\bullet+}$ (see text) at ZORA-(U)BP86/TZ2P, where the three-electron bonding interaction of the radical-cation Diels-Alder reaction is shown in red, the fragment orbital energies in the presence of the other reactant (black, in eV), gross Mulliken populations of fragment orbitals (blue, in electrons).

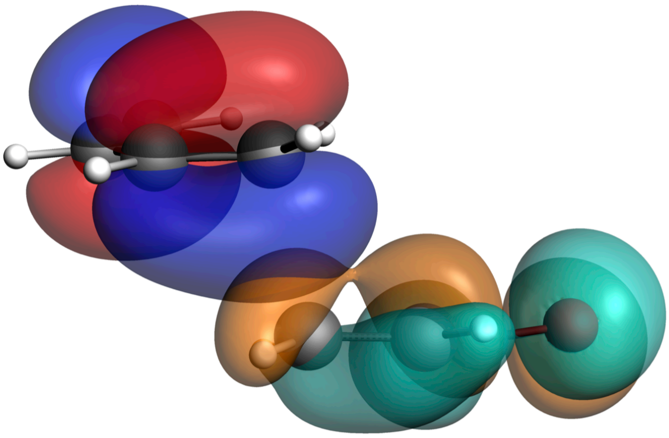

Figure S8. Representation of the overlapping π-HOMO$_{\mathbf{B}}$ and SUMO$_{\mathbf{O}^{\bullet+}}$ (isovalue = 0.03 Bohr$^{-3/2}$) at consistent TS-like geometries **BX** (*i.e.*, new C$_{\mathbf{B}}$•••C$_{\mathbf{X}\beta}$ bond distance is 2.203 Å for **X** = **O** and **O**$^{\bullet+}$; see text) at ZORA-(U)BP86/TZ2P.

Table S3. Cartesian coordinates (in Å), ADF total energies (in kcal mol^{-1}), and number of imaginary frequencies of all stationary points, computed at ZORA-(U)BP86/TZ2P.

1,3-butadiene (B)

E = -1293.71
H = -1238.39
G = -1258.36
N_{imag} = 0

C	1.372709	-0.713395	-0.499580
C	0.701805	-0.211221	0.547604
C	-0.701805	0.211220	0.547604
C	-1.372709	0.713394	-0.499580
H	0.889339	-0.878856	-1.463378
H	2.423105	-0.989233	-0.417892
H	1.222329	-0.125437	1.506398
H	-1.222329	0.125438	1.506398
H	-0.889339	0.878856	-1.463378
H	-2.423105	0.989233	-0.417892

acrylaldehyde (O)

E = -1079.86
H = -1039.30
G = -1059.22
N_{imag} = 0

O	-1.805437	-0.128643	0.000101
C	-0.681419	0.344494	0.000020
H	-0.520914	1.454121	-0.000072
C	0.558854	-0.450420	0.000033
C	1.757915	0.146949	-0.000061
H	0.445846	-1.536537	0.000121
H	2.689984	-0.416496	-0.000054
H	1.836090	1.236572	-0.000148

acrylaldehyde radical cation ($O^{\bullet+}$)

E = -852.19
H = -813.21
G = -833.27
N_{imag} = 0

O	-1.802570	-0.093405	0.000098
C	-0.655238	0.320151	0.000021
H	-0.566688	1.442208	-0.000070
C	0.568914	-0.465349	0.000034
C	1.758289	0.166247	-0.000062
H	0.436218	-1.548920	0.000123
H	2.679105	-0.422955	-0.000053
H	1.862889	1.252063	-0.000151

O-TS

E = -2361.37

H = -2264.59

G = -2291.35

$\mathbf{N_{imag}}$ = 1, ν = 391.095i cm^{-1}

C	-0.984945	0.969366	0.286069
C	-0.051477	1.632967	-0.508167
H	0.347730	2.584704	-0.159161
C	-2.044892	0.151645	-0.298464
O	-3.075323	-0.188802	0.272101
H	-1.866135	-0.126365	-1.370893
H	-1.112862	1.242784	1.335295
H	-0.144688	1.548578	-1.591315
C	0.171966	-1.246589	0.877796
C	0.854625	-1.463843	-0.292425
C	1.702813	-0.498494	-0.877605
C	1.864892	0.773308	-0.342725
H	2.486204	1.494146	-0.874279
H	-0.593770	-1.940668	1.219214
H	0.610925	-2.349423	-0.883038
H	2.062548	-0.687178	-1.891227
H	0.498726	-0.505853	1.602924
H	1.833905	0.915851	0.736306

O-P

E = -2409.61

H = -2309.97

G = -2335.91

$\mathbf{N_{imag}}$ = 0

C	-0.591977	-0.109794	-0.417378
C	0.740295	0.005527	-1.192978
H	0.554100	0.369258	-2.213001
C	-1.548123	-0.998101	-1.177552
O	-2.608033	-0.650478	-1.656805
H	-1.196486	-2.060835	-1.286574
H	-1.053708	0.885308	-0.334941
H	1.188224	-0.997325	-1.283325
C	-0.337115	-0.687748	0.991389
C	0.866950	-0.075970	1.657170
C	1.775085	0.665679	1.013421
C	1.719020	0.937080	-0.465611
H	2.724929	0.828288	-0.900588
H	-0.209329	-1.784891	0.929841
H	0.976170	-0.253073	2.729663
H	2.606766	1.101253	1.571714
H	1.438307	1.991085	-0.637629
H	-1.227981	-0.531377	1.619211

$O^{\bullet+}$-RC

E = -2180.34

H = -2089.95

G = -2119.78

$\mathbf{N_{imag}}$ = 0

C	-2.521556	1.243164	-0.150534
C	-1.674189	2.120847	0.403592
H	-2.004537	3.116861	0.695619
C	-2.078232	-0.094266	-0.537369
O	-2.729460	-0.978198	-1.031428
H	-0.939908	-0.317425	-0.337046
H	-3.573279	1.463580	-0.341886
H	-0.627471	1.873212	0.583247
C	0.619619	-1.324094	-0.070656
C	1.525500	-0.780712	-0.959160
C	2.613755	0.096979	-0.643630
C	3.051241	0.399183	0.613183
H	3.902184	1.062933	0.759488
H	-0.047022	-2.121905	-0.397793
H	1.411525	-1.042677	-2.013917
H	3.159034	0.512462	-1.493320
H	2.596704	-0.019889	1.511288
H	0.675813	-1.138367	1.002754

$O^{\bullet+}$-TS-1

E = -2176.98

H = -2081.02

G = -2108.79

$\mathbf{N_{imag}}$ = 1, ν = 243.350i cm^{-1}

C	-1.722851	-0.988967	-0.993391
C	-1.293029	-0.361235	0.158267
H	-1.419136	-0.866364	1.115464
C	-1.876363	-0.258273	-2.261679
O	-2.064133	-0.841583	-3.316480
H	-1.763162	0.850066	-2.225839
H	-1.887131	-2.067898	-1.035772
H	-1.228577	0.727420	0.180735
C	2.172539	1.996806	0.070669
C	1.846521	1.298648	1.197708
C	1.279767	-0.010133	1.230002
C	0.871184	-0.773669	0.135228
H	0.718492	-1.842273	0.272102
H	2.609995	2.991648	0.141896
H	2.060163	1.754105	2.166450
H	1.140633	-0.449673	2.220771
H	2.030174	1.597505	-0.933806
H	1.085156	-0.449997	-0.881919

$O^{\bullet+}$-INT-1

E = -2182.29

H = -2084.57

G = -2113.89

N_{imag} = 0

C	-1.498751	-1.052053	-1.150115
C	-0.997690	-0.444535	0.080861
H	-1.392829	-0.959857	0.968426
C	-2.100164	-0.289206	-2.229109
O	-2.471462	-0.838195	-3.264163
H	-2.206379	0.809580	-2.084462
H	-1.392021	-2.127452	-1.318439
H	-1.245378	0.622275	0.138260
C	2.389127	1.900877	0.111882
C	1.877724	1.329181	1.246428
C	1.109540	0.149119	1.270907
C	0.628128	-0.629927	0.142612
H	0.787525	-1.703568	0.312188
H	2.983958	2.812165	0.168559
H	2.090098	1.799167	2.208293
H	0.785020	-0.193503	2.258896
H	2.233039	1.479753	-0.881420
H	1.062318	-0.341218	-0.818946

$O^{\bullet+}$-TS-2

E = -2181.09

H = -2084.01

G = -2111.88

N_{imag} = 1, ν = 52.319i cm^{-1}

C	-1.498751	-1.052053	-1.150115
C	-0.997690	-0.444535	0.080861
H	-1.392829	-0.959857	0.968426
C	-2.100164	-0.289206	-2.229109
O	-2.471462	-0.838195	-3.264163
H	-2.206379	0.809580	-2.084462
H	-1.392021	-2.127452	-1.318439
H	-1.245378	0.622275	0.138260
C	2.389127	1.900877	0.111882
C	1.877724	1.329181	1.246428
C	1.109540	0.149119	1.270907
C	0.628128	-0.629927	0.142612
H	0.787525	-1.703568	0.312188
H	2.983958	2.812165	0.168559
H	2.090098	1.799167	2.208293
H	0.785020	-0.193503	2.258896
H	2.233039	1.479753	-0.881420
H	1.062318	-0.341218	-0.818946

O$^{\bullet+}$-INT-2
E = -2182.53
H = -2084.85
G = -2113.36
$\mathbf{N_{imag}}$ = 0

C	-0.805476	-0.363563	-1.215548
C	-0.873799	-0.914962	0.133804
H	-1.211537	-1.964694	0.060283
C	-1.564379	0.793772	-1.674147
O	-1.377302	1.267030	-2.793035
H	-2.290353	1.249683	-0.965567
H	-0.192480	-0.853842	-1.977215
H	-1.580573	-0.376495	0.777107
C	1.758488	1.177616	-0.723835
C	1.428583	1.315692	0.602568
C	0.947275	0.255861	1.383279
C	0.562262	-1.058868	0.869317
H	0.436127	-1.780840	1.681542
H	2.006865	2.045153	-1.335673
H	1.491902	2.303375	1.062913
H	0.708820	0.472668	2.428094
H	1.853106	0.203834	-1.202685
H	1.262713	-1.465287	0.129204

O$^{\bullet+}$-TS-3
E = -2182.45
H = -2085.54
G = -2112.94
$\mathbf{N_{imag}}$ = 1, ν = 210.436i cm^{-1}

C	-0.725702	-0.319007	-1.209981
C	-0.847035	-0.899109	0.126688
H	-1.216524	-1.934827	0.016056
C	-1.478881	0.845759	-1.677465
O	-1.369558	1.250666	-2.830456
H	-2.149978	1.343714	-0.942025
H	-0.142275	-0.837095	-1.977074
H	-1.558878	-0.351870	0.757281
C	1.640126	1.150195	-0.744231
C	1.377134	1.296147	0.598604
C	0.970181	0.230676	1.409255
C	0.552973	-1.081674	0.897032
H	0.398480	-1.797234	1.709533
H	1.807169	2.016624	-1.384839
H	1.427829	2.292266	1.042795
H	0.807001	0.435305	2.470543
H	1.800891	0.174449	-1.200834
H	1.267287	-1.508849	0.179522

O$^{•+}$-P
E = -2213.46
H = -2115.10
G = -2140.75
N$_{imag}$ = 0

C	-0.777632	-0.104170	-0.541574
C	0.323446	0.821664	-1.129533
H	0.014898	1.847351	-0.883990
C	-0.437584	-1.550190	-0.909891
O	-0.920557	-2.193159	-1.796501
H	0.375507	-1.987498	-0.250335
H	-1.755113	0.143186	-0.971331
H	0.333873	0.749934	-2.223087
C	-0.769353	-0.038735	0.991035
C	0.581876	-0.261172	1.554465
C	1.726653	-0.025901	0.822377
C	1.726179	0.534637	-0.551250
H	2.316321	-0.145567	-1.193552
H	-1.510034	-0.703309	1.460550
H	0.668872	-0.621357	2.580996
H	2.694333	-0.268624	1.267740
H	2.337491	1.454465	-0.547771
H	-1.112081	0.982331	1.267681
